# South American Physicians

**Published:** 1856-02

**Authors:** 


*South American Physicians.*—Dr. Hills—Dear Sir:—I take the liberty of sending you an extract of a letter from my friend Dr. M———, of Rio Janeiro, in Brazil, showing some of the beauties of medical practice in that country, which may prove of some interest to you as well as the readers of your Journal. G. A. Kunkler.

He remarks:—It is a mistaken idea which many have that practice of medicine in the South American States is very lucrative, and many who come to this country with no other object, often find themselves most grieviously deceived. I will therefore give you some particulars of a physician's life here.

His customary fee here, as in nearly all South American States, is one *patacon*, or 2000 reis, (about $1.30,) for each visit. This fee is stationary, and is charged by all. Night visits, smaller surgical operations, dressing of wounds, are all the same price, and only with extraordinary generous patients or in highly difficult cases, the fee is doubled. One laudable feature in our practice is that the payment is immediate, as soon as the visit is made; some of the wealthier classes occasionally make a regular contract for one year with the physician, which however rarely proves profitable to the latter. In speaking of visits, I must mention that only those in which a prescription is left are paid for; where only an examination is made, or regimen directed, or the use of former medicines ordered to be continued, no fee whatever is paid. In what a lamentable position the patient is placed by this custom, I need not stop to explain; as a natural consequence he is stuffed and flooded with remedies the physician and apothecary reaping the profit. The fee for an accouchement, (to which however physicians are seldom called,) is half an ounce of gold; in regard to larger surgical operations, it is customary to make a regular contract, and one-half of the stipulated fee is paid in advance; good security is demanded for the balance, or it will never be received. Surgery is chiefly in the hands of men (mostly Germans) who travel from one plantation to the other in quest of patients. This practice is the most lucrative, but has also its shady side, as it is not at all unfrequent that the gentlemanly planter relieves the surgeon of his fee, by lying in wait for him with a good knife or gun. Those however who are acquainted with this pleasant custom of the country, generally leave after the completion of the cure, without much ceremony, and escape the gratitude of the patient, by leaving post-haste in secret. To apply to the law for redress in nearly anything, is totally out of the question; partly on account of the miserable manner in which the law is administered, and partly on account of the enormous expense connected with it. In nearly every country it is necessary to remain stationary for some time to obtain practice; here it is different. During the first year you have a regular rush, during the second less, and in the third or fourth scarcely enough to live upon. Brazilian physicians, therefore, are shifting incessantly from one place to the other, and it is rare to find a man who has been in the place 8 or 10 years at a time.

For important competitors of physicians, we have here the various saints. If a patient recovers, Saint Anton, or Benedict, etc., etc., receive all the credit and incense; if he die, the whole responsibility falls upon the physician. As soon as a member of a family is taken sick, the *Cappellinha*, a small case containing a crucifix, with the virgin and divers saints, is brought to the patient, who then makes various promises of donations, to influence them in his behalf.

The superstition as well as the different irrational customs of the country, make the practice of physic very laborious. In entering the sick room, you are nearly suffocated by the fumes of incense, the odor of flowers, etc., which fill the room, (all for the honor of the patron saint.) The patient is always dressed and actually buried in a mass of bed clothing, doors and windows are carefully closed, and the apartment kept quite dark, and scarcely any light is allowed in the room, even while examining the patient. In being called to a female patient, it is considered an imperative duty to examine her abdomen, whether any thing be the matter with it or not, but the feet and other parts of the body are concealed with the utmost care.

Consultations are frequent, but never take place until the patient is in a dying condition. Instead of having them secretly, as with us, they are here considered as a kind of public exhibition; the apartment is therefore always crowded, and all the proceedings are public. The fee for a consultation is about $2 to $3 for each physician. Homeopathists are found occasionally, but are patronized indifferently, their treatment not being considered sufficiently energetic. Criminal abortions are very frequent, but the law rarely if ever interferes, although there are persons who make it their trade to produce them. All the general comforts of life command high prices.—*Medical Counsellor.*

